# Gigantism in a McCune-Albright’s syndrome with calcified GH-releasing pituitary adenoma: Case report and literature review

**DOI:** 10.1016/j.ijscr.2018.10.030

**Published:** 2018-10-23

**Authors:** Miguel Vega-Arroyo, Martha Lilia Tena-Suck, Celia Teresa de Jesús Álvarez-Gamiño, Citlaltepetl Salinas-Lara, Juan Luis Gómez-Amador

**Affiliations:** a Neurosurgery Department, National Institute of Neurology and Neurosurgery “Manuel Velasco Suárez”, Mexico City, Mexico; b Neuropathology Department, National Institute of Neurology and Neurosurgery “Manuel Velasco Suárez”, Mexico City, Mexico

**Keywords:** McCune-Albright’s, MAS, Gigantism, Pituitary, Adenoma, Calcification

## Abstract

**Background:**

McCune-Albright’s syndrome (MAS) is a rare disorder that is characterized by café-au-lait macules, fibrous dysplasia of the skull and endocrinopathies like excessive secretion of growth hormone by a hyper-functional pituitary adenoma (PA).

**Case:**

We describe the case of a 43-year-old male with history of Gigantism in 1990 secondary to a GH-secreting pituitary macroadenoma that was treated via microscopic transsphenoidal surgery at that time.

He was reported as asymptomatic for 26 years until he developed headache and right temporal hemianopia with left amaurosis. Also ptosis and proptosis was found caused by a re-growth of the tumor on the follow up MRI.

A second surgical procedure was performed via a dorsolateral craniotomy. Gross total resection was also achieved with a Neuropathology report of a pituitary adenoma tissue accompanied by extended dystrophic calcification and bone formation.

**Conclusion:**

This is a rare case of MAS. Gigantism within the context of a pituitary calcification raises special diagnostic and therapeutic challenges. The cause of the excessive secretion of GH in MAS is not well understood concluding that it seems to be a different etiology of patients with Acromegaly and Gigantism in non-MAS patients.

## Highlights

• There have been very few cases of pituitary calcified associated with McCune Albright syndrome.
• Pituitary calcification is quite rare by itself.
• Unlike this is a benign disease, its behavior is very aggressive and this highlights the need of a multidisciplinary team.
• It is mandatory to rule out a whole work up when McCune Albright syndrome is suspected to stratify risks and survival.

## 1 Introduction

McCune-Albright’s Syndrome (MAS) is a rare disorder characterized by café au lait macules, fibrous dysplasia of the skull and pituitary endocrinopathies like hyperfunctional pituitary adenomas with high plasma levels of Growth Hormone (GH) [1,2]. The hypersecretion of GH due to a pituitary hyperplasia or Pituitary Adenoma (PA) is a rare condition that only can be proven in 20–30% of patients with MAS [[3], [4], [5]]. On the other hand, the presence of a calcification in a PA is an uncommon feature that appears radiologically in 0.2–14% of the cases [6,7], and it has been slightly more common in the microscopic analysis, ranging from 5.4 to 25% [8,9]. Nevertheless, the presence of dystrophic calcification associated to PA is a very rare condition that has been described in 6.75% of the series [10], but never in the context of a MAS.

Identifying dystrophic calcifications in a PA in the preoperative imaging studies may represent a key role on the surgical decisión, since the scope in achieving a gross total resection can be more difficult to accomplish in the occurrence of extensive calcification [6,11]. Medical treatment has been the only option in MAS patients with GH excess mainly because the transsphenoidal surgery is quite and hardly restricted due to the massive thickening of the skull base [12].

We reported a rare case of a 43 year old male with gigantism due to an invasive PA in association of MAS that showed extensive dystrophic calcifications that calcified the pituitary tumor. The present case has been reported in accordance with the SCARE guidelines for case reports [13].

## 2 Case report

### 2.1 History and examination

A 43-year-old man with pituitary gigantism from the age of 16 secondary to a GH-functional PA. He underwent microsurgical trans sphenoidal surgery for resection of a PA in 1990 and adjuvant radiotherapy given in 2011 (radiotherapy scheme, original radiology and pathology unavailable). In 2016, almost 27 years after the initial surgery, he presented progressive decrease in vision on the left eye for six months. He also complained of excessive perspiration and sweating and had a past history of carpal tunnel syndrome. He was found to have gigantism features as extreme physical size (2.07 mts height) and pigmentation around eyes, neck and flexures.

**Fig. 1:**
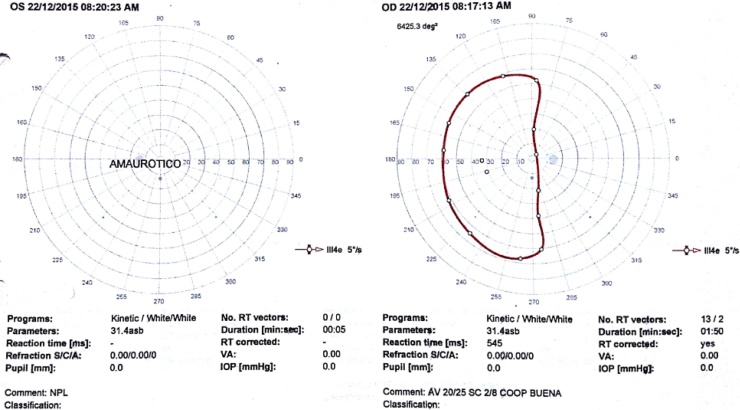
Goldmann’s perimeter test. Left amaurotic eye (*Left*) and right temporal hemianopia (*right*) (Octopus 900 Haag Streit Inc., Bern, Switzerland).

In ophtalmologic examination, a left ptosis with both exophthalmos and upward/inward limitation on left eye movements was achieved, and in the Goldmann’s test perimeter an ipsilateral amaurosis and right hemianopia (Octopus 900 Haag Streit Inc., Bern, Switzerland) (Fig. 1).

Random serum GH level was 0.071 ng/ml (0.003–0.97 ng/mL) with a normal GH concentration during the Oral Glucose Tolerance Test (OGTT) with a GH-nadir of 0.13 ng/ml (the gold standard for diagnosis is that GH excess fails to suppress serum GH level to less than 1 ng/ml after OGTT). The Insulin-like Growth Factor-1 (IGF-1) 84.3 ng/ml (64–210 ng/mL); the rest of hormones are listed and shown in Table 1.

**Table 1:** Pituitary work-up hormones. **TSH:** thyroid stimulating hormone, **T4:** thyroxine, **T3:** triiodothyronine, **LH:** Luteinizing hormone, **FSH:** follicle-stimulating hormone, **ACTH:** Adrenocorticotropic hormone, **GH:** growth hormone, **IGF-1:** Insulin-like growth factor 1.

Hormone	Result	Reference
TSH	1.54	0.34–5.60 μUI/ml
T4	7.6	8.11–17.25 pmol/L
T3	1.0	3.19–6.6 pmol/L
LH	0.12	2.4–12.6 mUI/ml
FSH	0.58	3.5–12.5 mUI/ml
TESTOSTERONE	0.32	2.8–8.0 ng/ml
PROLACTIN	6.8	2.6–13.1 ng/ml
CORTISOL	2.3	8.7–22.4 μg/dl
ACTH	8.0	4.7–48.8 pg/ml
GH	0.071	0.003–0.97 ng/ml
IGF-1	84.3	64–210 ng/ml

**Fig. 2:**
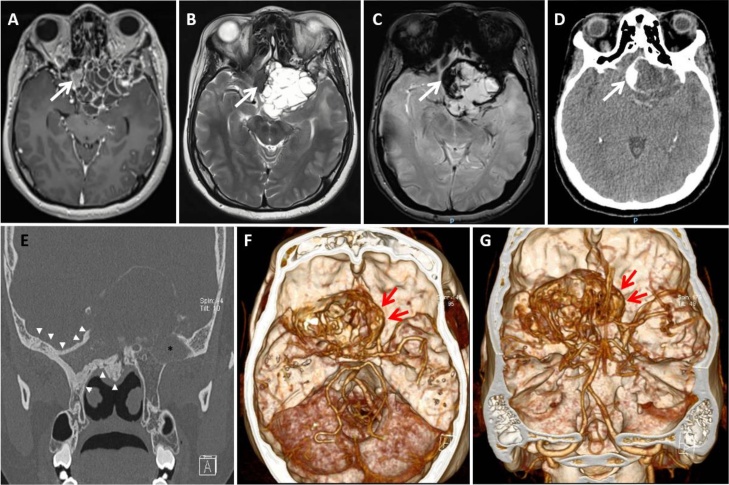
Preoperative Brain Magnetic Resonance-Imaging (MRI) and Computed tomography-scan (CT-scan). Axials (A) T1 + Gadolinium, (B) T2-weighted, (C) Gradient echo sequence with a large poli-cystic enhancing selar lesion with extensive osteophytic reaction and invasion of the ipsilateral orbital apex associated with fibrous dysplasia and an enhancing selar mass lesion with their respectively Calcium intensities (*white arrows*). CT-scans (D) axial CT-scan showing the calcified pituitary gland (*white arrow*) and (E) coronal on bone density revealing thickness of the diploe on the right sphenoid wing (*white arrow heads*) and osteolytic lesion on the left orbital apex (*black asterisk*). (F and G) axial and coronal 3D bone reconstruction showing calcified pituitary gland (*red arrows*) and a calcified rim around the tumor.

On brain Magnetic Resonance-Imaging (MRI) (Siemens 3.0 T magnetic resonance scanner and a 32-channel head coil) revealed a large policystic selar lesion with extensive osteophytic reaction and invasion of the ipsilateral orbital apex associated with fibrous dysplasia (see Fig. 2A and B) with a T2-weighted hypointense and enhancing selar mass lesion with Calcium intensity on the Gradient echo sequences (GRE) (Fig. 2C). Also the skull CT-scan (Siemens SOMATOM Sensation 64-slices) revealed an osteolytic lesion on the right orbital apex with thickness of the diploe (Fig. 2E) and pituitary gland with a calcified rim around the tumor (Fig. 2D, F and G). There was no personal or family history of prior endocrine disease.

### 2.2 Treatment course

**Fig. 3:**
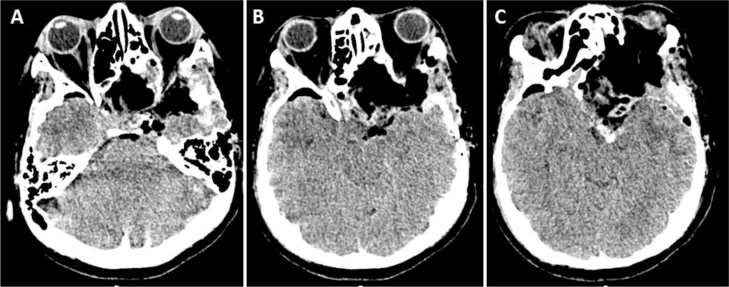
CT-scan. Axial slides (A – C) after tumor resection and post operative changes.

A second surgical resection was performed in 2016 by a left orbitozygomatic approach for tumor removal with no acute complications (Fig. 3A–C). Three days after the resection, the patient presented sudden unresponsiveness with an asymmetrically larger left pupil and rostrocaudal deterioration due to an ischemic stroke on left Anterior Cerebral Artery (ACA) and Middle Cerebral Artery (MCA), performing an urgent ipsilateral decompressive craniectomy. Unfortunately after surgery the patient continued unresponsive, leading to his death.

### 2.3 Neuropathology

**Fig. 4:**
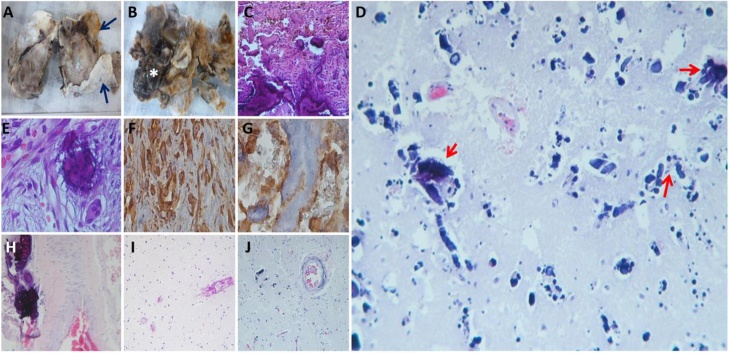
Gross aspect of the tumor (A) with outer greyish surface and thickened “like-capsule” (*blue arrows*) and (B) yellowish dystrophic calcifications alternating with areas of hemorrhage (*white asterisk*). Histological tumor features in (H&Ex400) (C) and (E) dystrophic calcifications of varying sizes and shapes. Immunohistochemical staining was positive for (F) growth hormone and (G). Histological basal ganglia features in (H&Ex800) (D) multiple calcifications on basal ganglia, (H) hypothalamus adjacent to the tumor, (I) brain tissue with fibrine inside vessel and (J) tortuous vessels con fine walls calcifications.

After the informant consent was obtained, brain autopsy was performed. Neuropathologic examination of the pituitary lesion removed in 2016 (first neuropathology report was unavailable) was characterized macroscopically by a 40 × 40 mm mass with grayish outer surface and inner yellowish-irregular zones thickened by “sand-like” calcifications (Fig. 4A and B). Histologically micro-hemorrhage with ossification areas within a thick pseudocapsule and multiple calcifications with a diffuse desmoplastic stromal component even within blood vessels were observed (Fig. 4C and D). Immunohistochemistry stains were positive for Growth Hormone and osteoconine (Fig. 4F–G). Brain autopsy showed perivascular granular-dystrophic calcifications in thalamus, hypothalamus and basal ganglia (Fig. 4D, H–J).

## 3 Discussion

The MAS has been thought as a consequence of postzygotic mutations in GNAS1, located on 20q13.3, a complex gene that uses several promoters to produce gene products like the ubiquitously expressed α-subunit of the Gs stimulatory protein (Gsα) [2,14]. This involving a couple hormones and seven-transmembrane receptors to adenylyl cyclase that facilitate the production of intracellular cyclic AMP leading to the receptor activation results in overproduction of hormones, manifested clinically as hyperfunctioning endocrinopathies and increased skin pigmentation [1,15,16]. MAS arise sporadically and there are no confirmed cases of vertical transmission.

Recently, some molecular and genetic etiologies of pituitary gigantism have been revealed to produce several genetic diseases like Multiple Endocrine Neoplasia (MEN) type 1 and 4, MAS, Carney complex, familial isolated PA or in association to defects in the familial succinate dehydrogenase-genes and the recently identified X-linked acrogigantism (X-LAG) syndrome [2,17].

The biological activities of Bone Morphogenetic Proteins (BMPs) in the endocrine system have been revealed. For example, the BMP-4 plays a key role in the initial development of the adenohypophysis and is also active and functionally involved in the differentiation of pituitary tumors, including prolactinomas and Cushing's disease but not in GH-releasing hormone PA [18].

Specifically in the primary pituitary tumors, one of the main neurosurgical cases that represents 10% of intracranial tumors and 10% in autopsy series as well [19,20]. The presence of proven calcifications in a PA has been scarcely reported with a prevalence rate of 0.2% to 14% in radiologic series [6,7] and ranges from 5.4 to 25% in histopathological cases [8,9]. Also some specific clinical features in PA, as hormonal markers and genetic variations, may predict the treatment course [9].

It is known that the presence of calcification in a PA is more frequent with prolactinomas, because the ossification derived from the osteo-metaplasia from the mesenchymal fibroblasts due to the effects from secondary ischemia by the tumoral outgrowth and the autocrine effects of prolactin hormone (Prl) [8,18].

Only eleven cases of a calcified PA have been described, comprising 5 reports with prolactinomas, 3 patients with a GH-secreting adenomas, 2 patients due to a so-called “TSHomas” and one patient with a gonadotrophin-secreting tumor [8]; but there are not previous reports associated to a MAS.

Additionally, matrix proteins are considered to be essential for biomineralization and to be important factors in cranioharyngioma calcification, like the Osteopontin (OPN) as a noncollagenous, acidic bone-matrix glycoprotein, which binds tightly to hydroxyapatite and appears to form an integral part of the mineralized matrix, probably important to the integrity of cell-matrix interactions [21].

Since the surgical management in craniofacial deformities is complicated in particular by the frequent post-operative FD re-growth, the treatment should be focused on the correction of functional deformities and notably in the optic nerve decompression in patients with objective vision loss [22]. Conversely, the decompression increases the risk of vision loss when is preserved, this last has been considered as contraindicated [1,23].

## 4 Conclusion

This is a rare case of gigantism with micro-calcifications in a GH-releasing PA associated to MAS. There has been a recognized association between Gigantism and MAS, however the presence of dystrophic calcifications in PA is rare and has to be recognized in the preoperative imaging as an important finding to consider on the surgical decision-making, since it may determine the complexity of its resection caused by struggling technical issues in the trans sphenoidal surgery due to thickening of the skull base [6,12]. Further investigations have to be done to clarify this rare disease.

## Conflicts of interest

All authors report no conflict of interest.

## Funding

None funding.

## Ethical approval

The internal review board of the National Institute of Neurology and Neurosurgery does not require ethical approval for case reports when informed consent was taken, if required by the journal it could be granted.

## Consent

Written informed consent was obtained from the patient for publication of this case report and accompanying images. A copy of the written consent is available for review by the Editor-in-Chief of this journal on request.

## Author contribution

Miguel Vega Arroyo: Writing the paper, analysis, design and reviewing literature.

Martha Lilia Tena-Suck: Data collection and data analysis.

Celia Teresa de Jesús Álvarez Gamiño: Translating, design and reviewing literature.

Salinas-Lara Citlaltepetl: Data collection and data analysis.

Gómez-Amador Juan Luís: Concept of paper, getting approval and interpretation.

## Registration of research studies

Not apply.

## Guarantor

Miguel Vega Arroyo.

## Provenance and peer review

Not commissioned, externally peer reviewed.

## Rights assignment sheet

All the authors have contributed substantially to the design, realization, analysis and presentation of the products of this work, in such a way that they have taken responsibility for it. Each author believes that this article represents the communication of a valid, true and ethical work, and each author has reviewed the content of this writing and has approved it for publication, this authorization being dictated by the author of correspondence, but whose responsibility is delegated to it, without conflicts, by each of the authors.

In addition, all authors attest to the precision with which the analysis were made, to the point where human error not noticed allowed to achieve. The authors declare that this work has never been published, either partially or totally, in another scientific journal, and that it is not being considered anywhere else for publication. On the other hand, there is no affiliation with any organization with a direct or indirect monetary or ethical interest with the substance of what is discussed in this scientific paper; therefore all the authors declare that there is no conflict of interest that affects the design and the report of the results of the study.

## Data availability

All relevant data are within the paper.

## References

[1] Robinson C., Collins M.T., Boyce A.M. (2016). Fibrous Dysplasia/McCune-Albright syndrome: clinical and translational perspectives. Curr. Osteoporos. Rep..

[2] Rostomyan L., Daly A.F., Beckers A. (2015). Pituitary gigantism: causes and clinical characteristics. Ann. Endocrinol. (Paris).

[3] Bhansali A., Sharma B.S., Sreenivasulu P., Singh P., Vashisth R.K., Dash R.J. (2003). Acromegaly With Fibrous Dysplasia: McCune-Albright Syndrome—Clinical Studies in 3 Cases and Brief Review of Literature. Endocr. J..

[4] Chanson P., Dib A., Visot A., Derome P.J. (1994). McCune-Albright syndrome and acromegaly: clinical studies and responses to treatment in five cases. Eur. J. Endocrinol..

[5] Premawardhana L.D.K.E., Vora J.P., Mills R., Scanion M.F. (1992). Acromegaly and its treatment in the McCune-Albright syndrome. Clin. Endocrinol. (Oxf.).

[6] Rilliet B., Mohr G., Robert F., Hardy J. (1981). Calcifications in pituitary adenomas. Surg. Neurol..

[7] Ogiwara T., Nagm A., Yamamoto Y., Hasegawa T., Nishikawa A., Hongo K. (2017). Clinical characteristics of pituitary adenomas with radiological calcification. Acta Neurochir. (Wien).

[8] Ibrahim R., Kalhan A., Lammie A., Kotonya C., Nannapanenni R., Rees A. (2014). Dense calcification in a GH-secreting pituitary macroadenoma. Endocrinol. Diabetes Metab. Case Rep..

[9] Tamaki T., Takumi I., Kitamura T., Osamura R.Y., Teramoto A. (2000). Pituitary stone—case report. Neurol. Med. Chir. (Tokyo).

[10] Al-Brahim N.Y.Y., Asa S.L. (2006). My approach to pathology of the pituitary gland. J. Clin. Pathol..

[11] Frara S., Maffezzoni F., Mazziotti G., Giustina A. (2016). The modern criteria for medical management of acromegaly. Prog. Mol. Biol. Transl. Sci..

[12] Christoforidis A., Maniadaki I., Stanhope R. (2006). McCune-Albright syndrome: growth hormone and prolactin hypersecretion. J. Pediatr. Endocrinol. Metab..

[13] Agha R.A., Fowler A.J., Saeta A., Barai I., Rajmohan S., Orgill D.P., Group S. (2016). The SCARE statement: consensus-based surgical case report guidelines. Int. J. Surg..

[14] Weinstein L.S., Shenker A., Gejman P.V., Merino M.J., Friedman E., Spiegel A.M. (1991). Activating mutations of the stimulatory G protein in the McCune-Albright syndrome. N. Engl. J. Med..

[15] Turan S., Bastepe M. (2015). GNAS Spectrum of disorders. Curr. Osteoporos. Rep..

[16] Piersanti S., Remoli C., Saggio I., Funari A., Michienzi S., Sacchetti B., Robey P.G., Riminucci M., Bianco P. (2010). Transfer, analysis, and reversion of the fibrous dysplasia cellular phenotype in human skeletal progenitors. J. Bone Miner. Res..

[17] Hannah-Shmouni F., Trivellin G., Stratakis C.A. (2016). Genetics of gigantism and acromegaly. Growth Horm. IGF Res..

[18] Otsuka F. (2013). Multifunctional bone morphogenetic protein system in endocrinology. Acta Med. Okayama.

[19] Molitch M.E. (2017). Diagnosis and treatment of pituitary adenomas: a review. JAMA—J. Am. Med. Assoc..

[20] Molitch M.E. (2014). Nonfunctioning Pituitary Tumors.

[21] Chauvet N., Romanò N., Meunier A.C., Galibert E., Fontanaud P., Mathieu M.N., Osterstock G., Osterstock P., Baccino E., Rigau V., Loiseau H., Bouillot-Eimer S., Barlier A., Mollard P., Coutry N. (2016). Combining cadherin expression with molecular markers discriminates invasiveness in growth hormone and prolactin pituitary adenomas. J. Neuroendocrinol..

[22] Gabbay J.S., Yuan J.T., Andrews B.T., Kawamoto H.K., Bradley J.P. (2013). Fibrous dysplasia of the zygomaticomaxillary region: outcomes of surgical intervention. Plast. Reconstr. Surg..

[23] Lee J.S., FitzGibbon E., Butman J.A., Dufresne C.R., Kushner H., Wientroub S., Robey P.G., Collins M.T. (2002). Normal vision despite narrowing of the optic canal in fibrous dysplasia. N. Engl. J. Med..

